# Educated Physicians

**Published:** 1872-03

**Authors:** 


### EDUCATED PHYSICIANS.

Among the medical men of the United States are found many of the most eminent in literature, science, philosophy, and history; and yet there are too many who are not masters of the elementary principles of any branch of learning. In the greed of gold, or in the ambition to have the greatest number of graduates, our medical schools have tarnished their fair fame in too many cases by bestowing degrees on unworthy and incompetent young men. No young man ought be allowed to enter a medical school unless he has a thorough classical education; because no young man should ever study medicine unless he has an abiding ambition to be at the head of the profession, to be an honor to it; but to reach such a point at this age of the world is not to be hoped for without the basis of a collegiate education. A man may be a good doctor without knowing Latin and Greek, but he cannot be a man among his peers with this great drawback.

It is greatly to the honor of Harvard University, at Cambridge, Massachusetts, that there are not as many medical students there this year as last, by over one hundred; and this deficit has arisen from the fact that the Faculty have raised the requirements of entry into the medical department so much higher than they were before, that the young gentlemen applying were not able to pass the examinations. It is greatly to be desired that this honorable example should be followed by every medical school in the country. The question should not be "Have you a diploma?" but "Do you understand your profession?" Meanwhile, it will not be long before the fact of a young physician graduating at Harvard will be considered as a guaranty that he understands his business, and is worthy of immediate and large patronage. For a year or two Harvard may lose in the number of her students, but not longer; for young men of real ability will naturally go there, and will give character to the institution. When they leave it to go among the people they will naturally send their own students there. But let not the great poet disgrace his chair by proclaiming that medicine has done more harm than good.

